# The Application of Convolutional Neural Networks (CNNs) to Recognize Defects in 3D-Printed Parts

**DOI:** 10.3390/ma14102575

**Published:** 2021-05-15

**Authors:** Hao Wen, Chang Huang, Shengmin Guo

**Affiliations:** 1 Department of Mechanical and Industrial Engineering, Louisiana State University, Baton Rouge, LA 70803, USA; hwen1@lsu.edu; 2 Department of Civil and Environmental Engineering, Louisiana State University, Baton Rouge, LA 70803, USA; chuan25@lsu.edu

**Keywords:** defect classification, defect detection, image segmentation, CNNs, YOLOv4, Detectron2, additive manufacturing, process control

## Abstract

Cracks and pores are two common defects in metallic additive manufacturing (AM) parts. In this paper, deep learning-based image analysis is performed for defect (cracks and pores) classification/detection based on SEM images of metallic AM parts. Three different levels of complexities, namely, defect classification, defect detection and defect image segmentation, are successfully achieved using a simple CNN model, the YOLOv4 model and the Detectron2 object detection library, respectively. The tuned CNN model can classify any single defect as either a crack or pore at almost 100% accuracy. The other two models can identify more than 90% of the cracks and pores in the testing images. In addition to the application of static image analysis, defect detection is also successfully applied on a video which mimics the AM process control images. The trained Detectron2 model can identify almost all the pores and cracks that exist in the original video. This study lays a foundation for future in situ process monitoring of the 3D printing process.

## 1. Introduction

Machine learning (ML) has been widely applied in many areas such as computer vision, general game playing, economics, data mining and bioinformatics [1,2,3,4]. Besides the mainstream artificial intelligence (AI) field, many experts are exploring using ML in their own fields, and materials science is one of the areas [5]. Deep learning (DL) is a family of ML methods that use multiple processing layers to learn data representations, and it has made new progress in the application of data-driven methods in the field of materials science [5]. For example, convolutional neural networks (CNNs), recurrent neural networks (RNNs) and deep coding networks have demonstrated capabilities in material detection, material analysis, material design and quantum chemistry [6,7,8,9]. Convolutional neural networks (CNNs) have been used in image analysis since the 1980s, and they are inspired by studying the brain’s visual cortex [10]. A CNN is a feedforward artificial neural network (ANN) which can accept images directly as an input of the network to avoid complex preprocessing procedures that are carried out in traditional image recognition algorithms [5]. The character of this model requires a large amount of parallel computation, which takes a long time when using central processing units (CPUs) alone. However, with the tremendous increase in the computation power of graphics processing units (GPUs), large CNNs can be trained in a reasonable amount of time. Due to the increase in computation power and available data, CNNs have achieved superior performance on many complex visual tasks, such as image searches, self-driving cars, automatic video classification and more [5,10,11]. In recent years, many fundamental architectures have been developed, such as the LeNet-5 architecture (1998), AlexNet (2012), GoogLeNet (2014) and ResNet (2015), which have reduced the error rate from over 26% to only 3% in the ILSVRC ImageNet challenge [10,12]. As a result, CNN-based models can be very useful tools to analyze materials science-related images.

Nowadays, metal-based additive manufacturing (AM) processes, such as laser powder stream, or LENS^®^-Laser Engineered Net Shaping^TM^, electron beam wire feed, electron beam powder bed and laser beam powder bed fusion (LPBF), are widely used in industries. For making critical components, a fundamental requirement is to reduce/eliminate defects, such as pores and cracks, in the metal-based AM parts. In a laser-based AM process, pores are generally formed due to lack of fusion or keyholes, and cracks are usually associated with freezing contraction and microsegregation. As metal-based AM is a complex process, and many conditions cannot be controlled during printing, defects are hard to avoid initially, especially for some newly developed alloys. Since an AM process forms the parts layer by layer, fast and precise cameras can be added to 3D printers to generate in situ monitoring images or videos. It is envisioned that future in situ monitoring systems could automatically detect and classify defects and pinpoint defect locations during the AM process. Therefore, AM processing parameters, such as different laser scan speeds, scan patterns and laser powers, can be modified for the next layer to mitigate the defects formed in the previous layer. As a result, automatic detection and classification of defect types are becoming a prevalent task for improving metal-based AM processes.

The purpose of this paper is to demonstrate the capability of different CNN models for detecting and classifying cracks and pores, the two common defects in LPBF parts. The material image analysis in this study was carried out at three different levels of complexity. Level one is defect classification using a simple CNN, and the goal is to classify a defect in the image as either a crack or pore. A simple CNN consisting of three convolutional layers and two dense layers was used. Level two is target detection, and the model is designed to detect cracks and pores on a typical scanning electron microscope (SEM) image and draw bounding boxes around the defects. The outcome of level two is the capability to provide defect location and type information for advanced AM processing control. The selected model for target detection is YOLOv4, which is the latest variant (fourth version) of a popular object detection algorithm YOLO—You Only Look Once [13,14]. This target detector is faster and more accurate than the other available detectors such as LRF, SSD and M2Det. In addition, it can be trained on conventional GPUs with 8–16 GB-VRAM, which makes its broad use possible [13]. Level three is image segmentation. Besides providing the second-level information, the level three model should also generate information regarding the defects’ shape, which can be used for further physics-based simulations. The selected level three model for image segmentation is Detectron2. It is a Facebook AI Research (FAIR) software system that implements state-of-the-art object detection algorithms, including Faster R-CNN, Mask R-CNN, RetinaNet and Densepose [15,16,17]. Detectron2 is a newer version of Detectron, and it is implemented in Pytorch with a more modular design [15]. It has become the most widely used open source project of FAIR because of its enhanced flexibility and extensibility [15]. Finally, the Detectron2 model is also applied on a video which was created by a sequence of X-ray computed tomography (CT) images to test the capability of in situ monitoring. The CT technique is widely applied in the AM field for nondestructive evaluation of 3D-printed parts [18]. This study can potentially provide a tool for detecting defects in LPBF AM parts in situ, which can further offer a new perspective to eliminate cracks and pores by changing scan strategies during the subsequent layer of the LPBF printing process.

## 2. Materials and Methods

### 2.1. The Structure of Different Models

CNNs usually consist of convolutional layers, pooling layers, and fully connected layers. The neurons in the first convolutional layer are only connected to their corresponding receptive fields, and the neurons’ weights can be represented as a filter. Each filter will construct a feature map, and all the feature maps will be combined together to form a convolutional layer. This architecture allows the network to focus on low-level features and then assemble them into higher-level features in the next hidden layer [10]. The pooling layer is added to reduce the computational load by reducing the image size. At the end of the convolutional layers, all the feature maps will be flattened and followed by several fully connected layers, and the last layer outputs the prediction results.

YOLOv4 is a combination of a series of computer vision techniques which mainly consists of three parts: (1) a backbone: CSPDarknet53 [19]; (2) a neck: spatial pyramid pooling (SPP) [20], path aggregation network (PAN) [21]; and (3) a head: YOLOv3 [22]. In the backbone stage, the image is taken as input and goes through a CNN to extract features. YOLOv4 uses CSPDarknet53 as the backbone, which is developed based on DenseNet [23]. CSPDarknet53 can separate the feature maps into two copies; one copy goes to the dense block and the second copy can go directly to the next stage. This unedited version of the feature map can remove the computational bottlenecks [19,23]. The role of the neck stage is to mix and combine feature maps from different stages of the backbone [23]. The neck consists of an SPP block and a PAN. The SPP block can generate a fixed-length output with the most important features, and the PAN can achieve better propagation of layer information from bottom to top or top to bottom [22,23]. The final head stage will perform the last dense prediction, including the predicted bounding box’s coordinates, the confidence score and the classification label [22].

Taking Base R-CNN with feature pyramid network (Base-RCNN-FPN) as an example, the structure of Detectron2 mainly includes three parts: backbone network, region proposal network (RPN) and ROI head (box head). The backbone network can extract multi-scale feature maps with various receptive fields from the input image; the RPN can detect the object regions from the multi-scale feature maps, and by default, it will output a thousand proposed boxes with a confidence score; lastly, the box head can use the proposal boxes to crop the feature maps into different sized features, and through fully connected layers, it can find out the box locations and the classification labels [24]. The above functions are achieved through different classes in each stage. For example, FPN and ResNet are the classes within the backbone network; the RPN stage includes StandardRPNHead and RPNOutput classes; and the ROI head includes ROIPooler, FastRCNNConvFCHead, FastRCNNOutputLayers and FastRCNNOutputs classes [24].

### 2.2. Image Data Preparations

Using CNNs for LPBF AM, defect detection and classification often involve three subtasks: (1) collecting images that include cracks and pores, (2) manually/semi-automatically creating labels for a number of images, (3) training different CNN models using various techniques with the labeled images [25]. These subtasks are tackled at three levels of different complexity in this paper.

**Figure 1:**
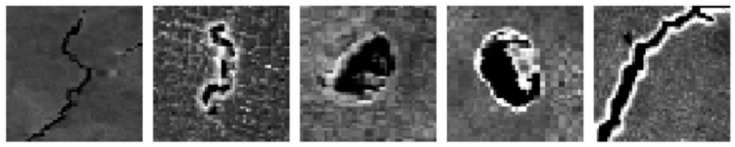
Examples of training data for the level one CNN model.

For level one defect classification, a total of 200 images, with a single defect in each image, were collected as screenshots from SEM images of LPBF-processed Ni939 samples, which contain multiple cracks and pores. Therefore, there is only one defect that needs to be classified on each of these generated images. Two file folders were created (“Cracks” and “Pores”) to include the single-defect images, and the folder names act as the defects’ class labels. Additionally, 5 images in each category were used as validation data to test the model accuracy during the training process. A simple CNN model was trained using these images to distinguish between cracks and pores. Figure 1 shows some example images of the training data.

**Figure 2:**
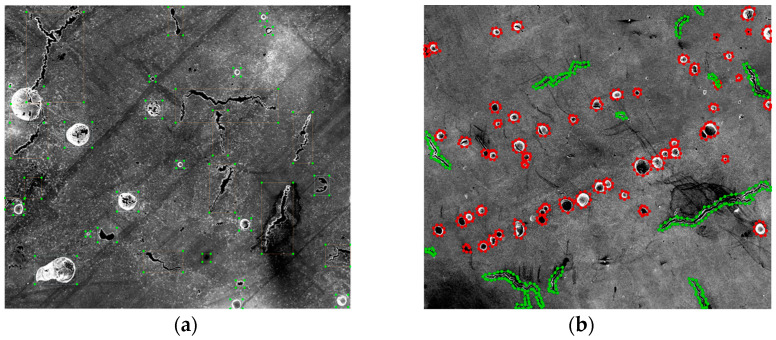
(**a**) An example of the labeled image using LabelImg for training YOLOv4. (**b**) An example of the labeled image using Labelme for training the Detectron2 model.

For target detection and image segmentation at level two and level three, SEM images of AM parts were used. A total of 11 SEM images, taken on LPBF-processed Ni939 samples, were collected from our SEM image archive. Among the 11 images, nine images were used as training, and the remaining two images were used as testing. According to the common practice, there are two image annotation software packages commonly used for labeling the data. The cracks and pores on these images were manually labeled using LabelImg, which can only draw the bounding box around the defects [26]. These labeled images were then used for training YOLOv4. The second software is Labelme, an image annotation tool that can outline the shape of the cracks and pores [27]. These labeled images were used for training Detectron2. Figure 2 shows an example labeled image using LabelImg and Labelme.

For defect detection in video files, about 1800 cross-section CT scan images, obtained on an LPBF Ni939 sample, were accumulated using 3D X-ray microscopy (Zeiss XRM 620 Versa, White Plains, NY, USA). Due to the size limitation, a sequence of 100 images was imported to ImageJ software to generate a 14-s video with a frame rate of 7 fps. This video was then used for testing the trained Detectron2 model. Ten different images were chosen from the above CT scan images to generate the training data. The training data were manually labeled with cracks and pores with the same process as image segmentation using Labelme software.

### 2.3. Training Process for Different Models

For training the level one CNN model, the images needed to be imported to the model at the same size to improve the training accuracy. As a result, the 200 single-defect images were randomly shuffled and imported to Python at the same size of 100 × 100 pixels. Ten images (five images for each category) were used as test data to validate the model’s performance. The codes were generated using Python in Jupyter Notebook, and the ML packages included os, Numpy, Matplotlib and Tensorflow. All the images were imported to the CNN model with the label of either “Cracks” or “Pores”, and the model was built for binary classification. The CNN model has three convolutional layers, each followed by a maxpooling layer. Two dense layers are added at the end, and the activation functions are “relu”. “Adam” is used as the optimizer and the loss function is “BinaryCrossEntropy”. Table 1 shows a summary of the model (this table is reformatted based on the original code output, and the direct code output table is provided in the Supplementary Materials). The model was trained for 20 epochs with training and testing accuracy tracked during the training process.

For training the YOLOv4 model, the darknet was cloned and rebuilt from AlexeyAB’s repository to Google Colab [28,29]. As shown in Figure 2a, the labeled image contains the defect bounding box. The coordinates of the bounding box and defects’ category information were included in a .txt file for each image. The yolov4-custom configuration file was adjusted based on two classes, and the training parameters included batch = 64, subdivision = 16, max_batches = 3600, steps = 2880, 3240, width = 416 and height = 416. The classes were set to two for the three YOLO layers, and the filters = 21 in the three convolutional layers before the YOLO layers [30]. The pre-trained weights for the convolutional layers were loaded to the YOLOv4 network before training to achieve higher accuracy and shorten the training time. The model was trained for 3600 iterations, and the best weights were stored in a Google Drive for later validation.

For training the Detectron2 model, a total of 828 pores and 301 cracks were manually labeled from the nine SEM images using Lableme software. As shown in Figure 2b, it can be found that the shapes of the defects are outlined with a different color. These images contain instance and shape information compared with the YOLOv4 model. These detailed data can be exported to other software for further analysis and simulations, such as finite element analysis (FEA) for crack propagation predictions under specific load conditions. The codes were generated using Python in Google Colab, and the ML packages included Pytroch, Detectron2, OpenCV and Numpy. Firstly, the labeled data were converted to coco format and registered as coco instances. Model Mask-RCNN-R50-FPN was chosen from the Detectron2 Model Zoo. The other training parameters included image_per_batch = 2, Base_learning_rate = 0.0025, training_iterations = 15,000 and batch_size_per_image = 512. The training curves were tracked using Tensorboard during the training process. After training, the remaining two images were used to test the performance of the model.

For the video testing, the Detectron2 R101_DC5_3x model was trained using Google Colab for 5000 iterations with a learning rate of 0.0025. All other parameters were the same as above. Before testing on the video, the weight path in the configuration file was changed to the trained model’s final weight; the test threshold was set to 0.3; and the detection per image was increased to 1000 per image.

## 3. Results and Discussion

**Figure 3:**
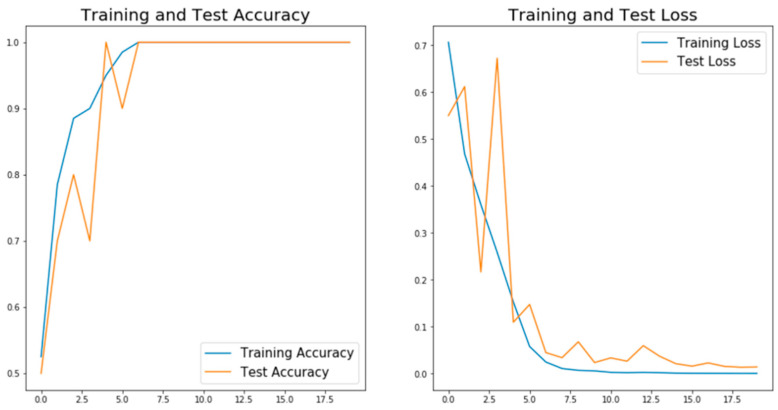
The accuracy and loss of training and validation for the level one CNN model.

**Figure 4:**
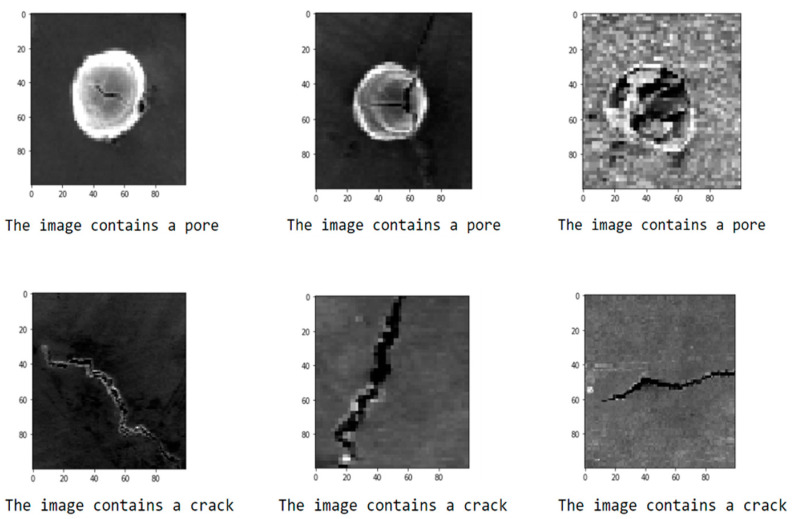
The test result for the level one CNN model using six unknown data.

Figure 3 shows the “accuracy” and “loss” changes with epochs for the level one CNN model. The training and validation accuracies almost reach 100%, and the loss is nearly 0 only after ten epochs of training. Such perfect outcome usually indicates that the model is overfitting, which means the model achieved high accuracy by just simply remembering each image category. Overfitting is a very common phenomenon, especially for cases with a small dataset [31]. A model with overfitting typically cannot generalize well on the new data. To validate whether overfitting exists in our model or not, six new test images that the model never saw during training were imported to the CNN model to test if it could classify the defects correctly. Figure 4 shows the classification results, and all the defects are classified correctly. For these new images, the model performs perfectly and achieves very high accuracy. Based on the fact that all the training, validation and new test data are evenly distributed, it can be concluded that the model is not overfitting. The high accuracies are achieved mainly because this is a relatively simple dataset with obvious signals for each category. The pores are round-shaped, and the cracks have a long and narrow line shape, which can be easily distinguished. Another possible reason for this superior performance is that all the data are manually selected without any mistakes and confusion images, which can minimize the errors during the training and validation process.

**Figure 5:**
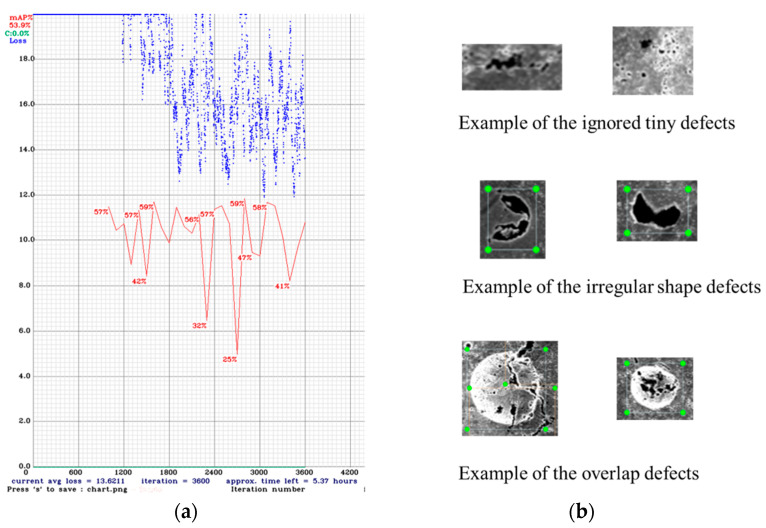
(**a**) The total loss and AP curve of YOLOv4 training process (the *X*-axis is the training iterations, the *Y*-axis is the total loss, the blue curve is the total loss and the red curve is the AP). (**b**) Examples of the data complexity.

The YOLOv4 model was trained using the Google Colab Tesla P100-PCIE-16GB GPU for 3600 iterations, and the best weights were generated by the code and autosaved in a Google Drive. The final total loss reached around 16, and the average precision (AP) achieved about 50%. Figure 5a shows the total loss and the AP curve recorded during the training process. It can be found that the total loss value indicated by the blue line falls below 20 after about 1200 iterations of training. After this, the loss value starts to bounce between 20 and 12, with tiny drops with the increase in training iterations. This indicates that the model has already reached the local minimum. Even the training keeps going, and there will not be an obvious drop for the total loss value. However, the loss value is still very high compared with other applications in which the loss value can drop to less than 2 [30,32]. After 1000 iterations of training, the AP value (red line) has been tracked for every 100 iterations of training, with its value marked above the line. Between 1000 and 3600 iterations of training, the AP value does not have a noticeable increase. On the contrary, it dropped few times, indicating the model already has some degree of overfitting. As a result, the training process can have an early stop. However, around a 50% AP value is deficient compared with some other applications [30,32,33,34]. The high loss and low AP value are most likely because of the complexity of the training data. (1) Some of the tiny features are ignored when labeling the image since there are too many, and it is impossible to label all of them. (2) Some of the defects have an irregular shape that is different from most of the other defects. (3) There are some overlap defects, which can cause confusion to the model. Some examples of the above data are represented in Figure 5b. These complexities can make the model hardly fit all the data; therefore, the total loss and AP had no improvements after about 1000 training iterations. Additionally, the relatively small training dataset size also contributes to the quick convergence of the training process.

**Figure 6:**
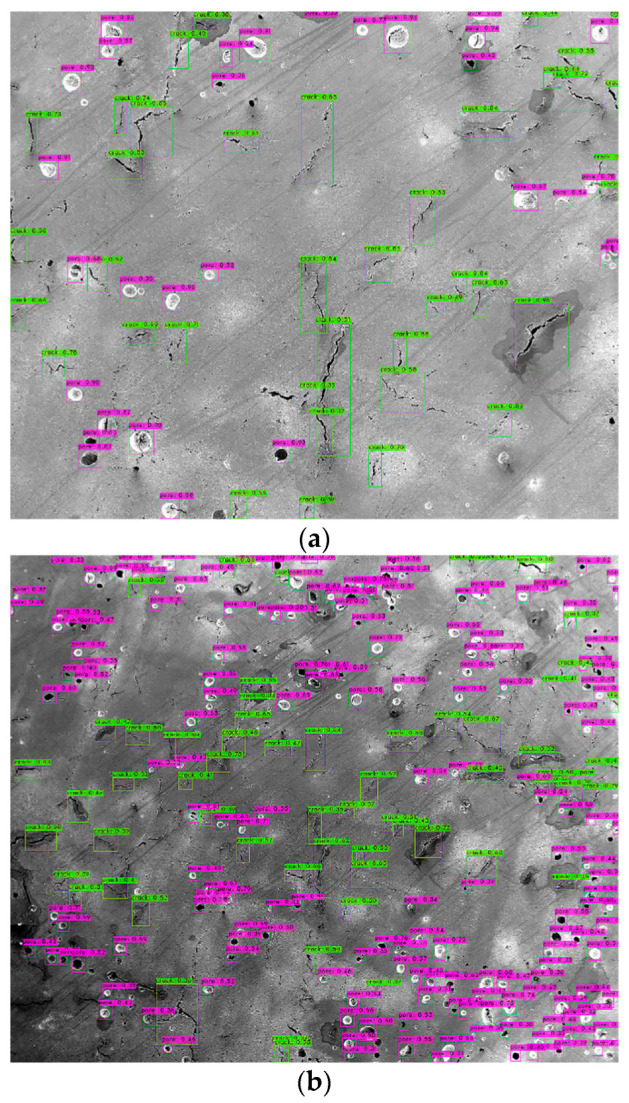
The test result of the YOLOv4 model with the best weights and threshold of 0.3 (the red arrows indicate some of the missed defects) on two images with different defect densities. (**a**) test image with less defect density (**b**) test image with higher defect density.

Before testing on new images, the configuration file was set to batch = 1, subdivision = 1 and threshold = 0.3, in order to test on a single image. Figure 6 shows the test results of two images with different defect densities. The bounding box, the defect category and the confidence score are marked on the image. It can be found that some defects are not recognized by this model. Some examples of the missed defects are marked with a red arrow in Figure 6a. By just visualizing the test result, the trained YOLOv4 model can generate acceptable results with about a 90% recognition rate. The result is calculated using the total number of the model-detected defects divided by the total defects that are included in the ground truth image. The above testing result only covers one set of parameters in the configuration file; however, many parameters may affect the final test results. Among them, the initial learning rate (LR) and scales are two important parameters. The LR needs to be high at the beginning of the training process with little knowledge of the features; however, the LR needs to be lowered with the increase in iterations. The scales are the factors to multiply the LR after the specified step numbers [32]. Since the model had little improvements after about 1000 iterations of training, max_batches was set to 1000 and the steps were set to 800 and 900, which can save training time. Table 2 shows the comparison of testing results with different LRs and scales. Among them, IoU stands for Intersection over Union, which computes the area of intersection over the union area of the ground truth bounding box and the predicted bounding box. It acts as the threshold to determine true positives (TPs) and false positives (FPs). When the model fails to detect an object, which is present in the ground truth image, it will be classified as a false negative (FN). Below are the equations for calculating average precision (AP) and recall values [35].

(1) $$\mathrm{AP}=\frac{1}{N}\sum^{\;}\frac{\mathrm{TP}}{\mathrm{TP}+\mathrm{FP}}$$

(2) $$\mathrm{Recall}=\frac{1}{N}\sum^{\;}\frac{\mathrm{TP}}{\mathrm{TP}+\mathrm{FN}}$$

**Figure 7:**
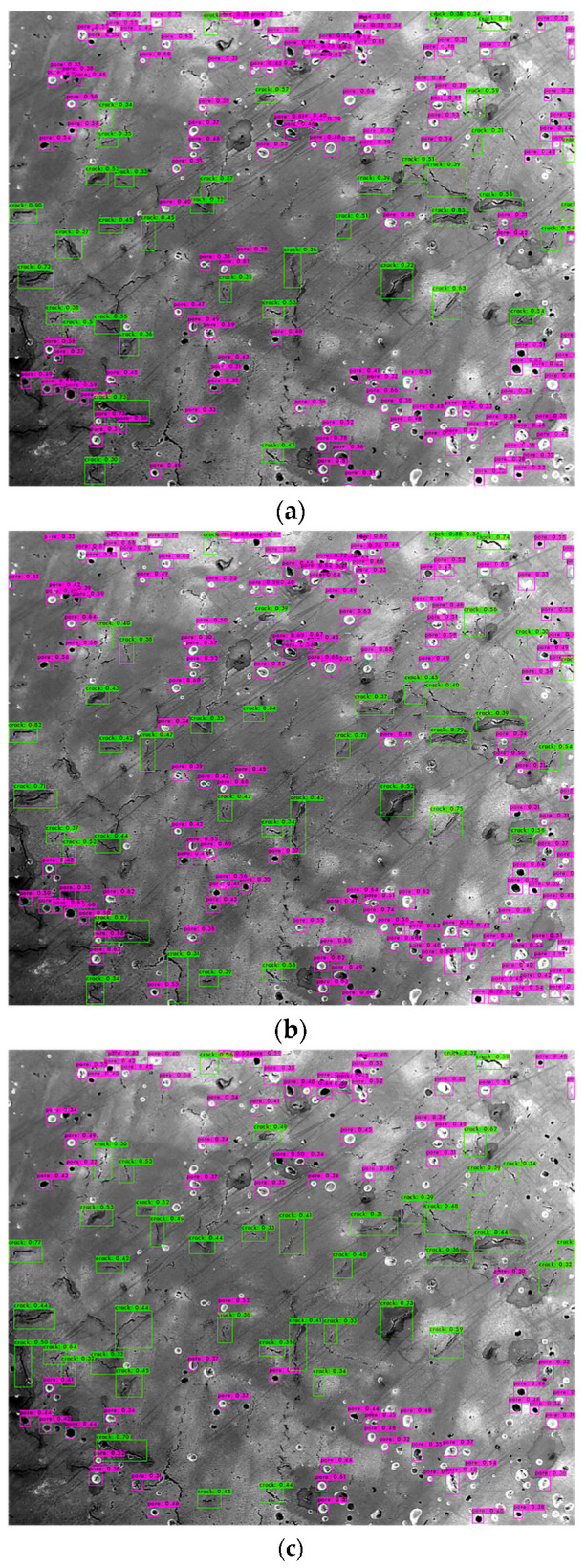
The testing results on the same image using trained YOLOv4 model with (**a**) No. 6 parameters, (**b**) No. 5 parameters and (**c**) No. 1 parameters.

Basically, N is the total number of classes. The AP value describes the accuracy of the predicted objects, and the recall value indicates the completeness of the predicted results. For both of these two values, the higher, the better. As a result, the No. 6 parameters achieved the best results. Figure 7 shows a comparison of the testing results on the same image using (a) No. 6, (b) No. 5 and (c) No. 1 parameters. In Figure 7b, more detections are made. However, these include many FP detections, which drops the AP to only 73%. Figure 7c has less detections compared with (a), but it cannot cover all the defects included in the ground truth image. Therefore, the recall value for No. 1 is only 0.35.

**Figure 8:**
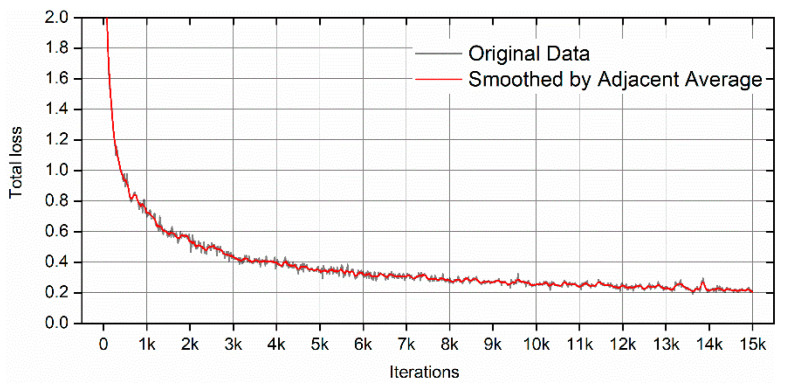
The total loss curve of Detectron2 during the training process. (The link of original output image from TensorBoard is provided in the Supplementary Materials).

**Figure 9:**
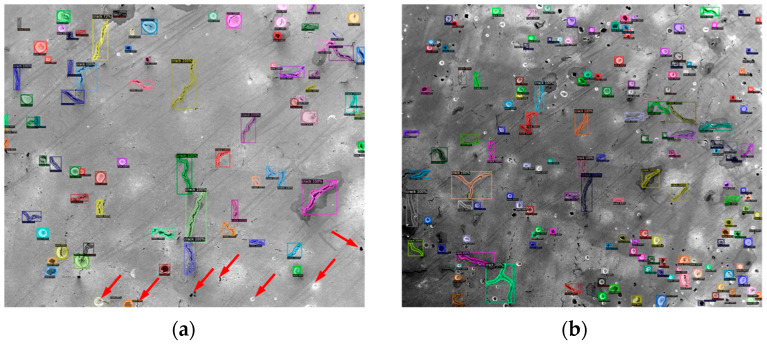
The test result of the Detectron2 model with the threshold of 0.3 (the red arrow indicates some of the missed defects) on two images with different defect densities. (**a**) test image with less defect density (**b**) test image with higher defect density.

The Detectron2 model was trained using the Google Colab Tesla P100-PCIE-16GB GPU for 15,000 iterations. The total loss value was tracked using Tensorboard, and the curve is shown in Figure 8. It can be seen that the total loss value drops with the increase in training iterations and finally reaches about 0.2. However, the total loss number cannot indicate model performance on the new data since it may have overfitting. Therefore, testing the trained model on the new images is necessary. Before testing, the testing threshold was set to 0.3, and the detections_per_image was set to 1000. This is used to cover a large number of defects in the testing images. Figure 9 shows the testing results using the trained Detectron2 model. The image used for testing is the same as that used for testing the YOLOv4 model. However, besides the bounding box, the result outlines the defects’ shape, which is extra information compared with YOLOv4. Similarly, there are also a few missed defects, which are marked in the red arrows in Figure 9a. By just visualizing the testing results using the Detectron2 model, the trained model can identify more than 90% of the defects. The result is calculated using the same method as the YOLOv4 model. However, for some more complicated images with a much higher defect density, the testing result of the Detectron2 model is not as good as the YOLOv4 model. By comparing Figure 6b and Figure 9b (both with the test threshold of 0.3), it can be found that the Detectron2 model missed more defects compared with the YOLOv4 model. Nevertheless, the Detectron2 model only takes about 3 h to train 15,000 iterations, and the YOLOv4 model takes more than 9 h to train 3600 iterations using the same GPU. Therefore, the Detectron2 model is more efficient than the YOLOv4 model based on the training process.

**Figure 10:**
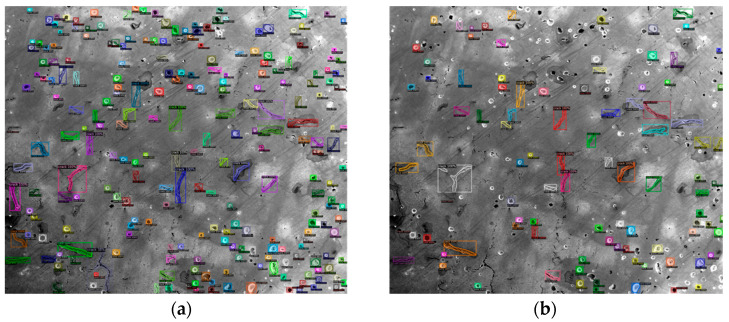
The testing results on the same image using (**a**) No. 3 model and (**b**) No. 1 model.

The Detectron2 Model Zoo has different baseline models, which are combinations of different backbones and training schedules [36]. Three models were selected to compare the performance of different baselines, and all the evaluations were performed using the same images as used in the previous testing. The results are shown in Table 3. Among them, the mAP value is the average AP for IoU from 0.5 to 0.95, with a step size of 0.05. AP50 is the AP with IoU = 0.5, and AP75 is the AP when IoU = 0.75. AR10 is the average recall value given 10 detections per image. Similarly, AR100 and AR1000 are the average recall values when the maximum detection per image is 100 and 1000, respectively. It can be found in Table 3 that for all three models, the AP and AR values only have minor variations with the increase in training iterations from 5000 to 10,000. This indicates that all the models will converge quickly because of the small training dataset. When the IoU threshold becomes larger, the AP value drops dramatically, meaning that the mAP value is low compared with YOLOv4. The AR value reaches the highest when the maximum detection per image reaches 1000. However, when the number is small, the AR value decreases significantly. Based on the mAP and AR1000 value, the No.3 model with 1000 iterations of training has the best testing result, and the No. 1 model with 5000 iterations of training is the worst among Table 3. Figure 10 shows a comparison of the testing result images between the best and worst models. By just visualizing, the conclusion is that the No. 1 model misses more defects than the No. 3 model.

**Figure 11:**
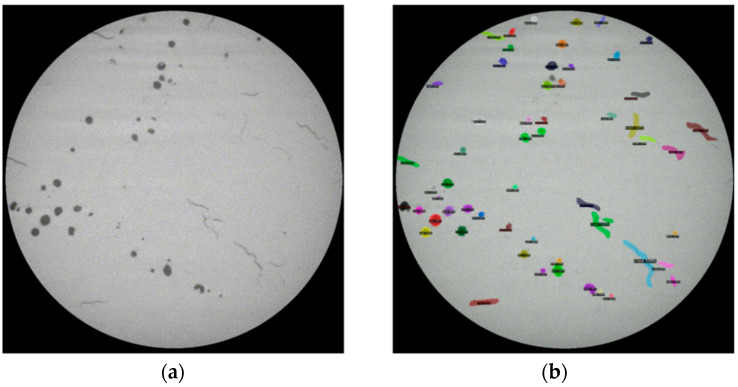
Comparison (the screenshot of the same frame) of the (**a**) original video and the (**b**) output video of the defect detection using the trained Detectron2 model.

For the testing on video files, a comparison of the original video frame and the output video frame after defect detection using the trained Detectron2 model is shown in Figure 11. (Due to the size limitation, Figure 11 is taken as a screenshot of the same frame of the original and the output video. The video files are provided in the Supplementary Materials.) It can be found that the trained Detectron2 model can detect almost all the pores and cracks which exist in the original video. Furthermore, the detections also present the defect category, defect location and the defect shape information in a very short period of time for each frame (based on the evaluator function that is included in Detectron2, it only takes about 0.22 s to generate the above information for each frame). This information can be beneficial for in situ AM process control. For example, the AM machine can be modified in real time to eliminate the defects by changing scan parameters or scan strategies when processing the following layer. However, due to the limitation of the current facilities and resources, further work regarding in situ AM process control needs to be performed in the future.

## 4. Conclusions

Three different levels of complexities—defect classification, defect detection and defect image segmentation—were successfully performed using a simple CNN model, the YOLOv4 model and the Detectron2 object detection library, respectively. The tuned simple CNN model can accurately classify cracks and pores. The YOLOv4 and Detectron2 models can identify more than 90% of the defects in the testing images. Defect detection was also successfully applied on a video file, generated using a sequence of CT scan images. The trained Detectron2 model can identify almost all the pores and cracks that exist in the original video. This capability lays a foundation for future in situ process control for 3D printers.

Overall, the authors demonstrated the application of deep learning models for materials science-related image classification, target detection and image segmentation tasks. This paper demonstrates that ML can be a powerful tool to support materials science research. However, the functionality of the ML models should be further improved with more data. Building a database and collecting more data closely related to the research purpose must be tackled to enable the application of ML in the field of materials science. For future AM process monitoring applications, it is envisioned that image streams obtained from high-quality cameras would be fed into the ML-based in situ monitoring system to automatically detect and classify defects and pinpoint defect locations during the AM process. This information will then be used to adjust AM processing parameters, such as different laser scan speeds, scan patterns and laser powers, for the next layer to mitigate the defects formed in the previous layer.

## Figures and Tables

**Table 1:** The summary of the level one CNN model.

Model.Summary()		
Model: “Sequential_3”		
Layer (type)	Output Shape	Param #
conv2d (Conv2D)	(None, 100, 100, 16)	160
max_pooling2d (MaxPooling2D)	(None, 50, 50, 16)	0
conv2d_1 (Conv2D)	(None, 50, 50, 32)	4640
max_pooling2d_1 (MaxPooling2D)	(None, 25, 25, 32)	0
conv2d_2 (Conv2D)	(None, 25, 25, 64)	18,496
max_pooling2d_2 (MaxPooling2D)	(None, 12, 12, 64)	0
flatten (Flatten)	(None, 9216)	0
dense(Dense)	(None, 512)	4,719,104
dense_1 (Dense)	(None, 1)	513
Total params: 4,742,913		
Trainable params: 4,742,913		
Non-trainable params: 0		

**Table 2:** The comparison of testing result with different LRs and scales.

No.	LR	Scales	AP	Recall	TP	FP	FN	Average IoU
1	0.001	0.1, 0.1	77%	0.35	150	46	274	54.16%
2		0.2, 0.2	79%	0.49	208	56	216	54.44%
3		0.3, 0.3	75%	0.49	207	69	217	52.19%
4	0.0005	0.1, 0.1	79%	0.47	199	52	225	56.34%
5		0.2, 0.2	73%	0.48	205	77	219	49.48%
6		0.3, 0.3	80%	0.5	210	51	214	57.19%

**Table 3:** The comparison of testing results with different models and iterations.

No.	Model	Iterations	mAP	AP50	AP75	AR10	AR100	AR1000
1	X101-FPN 3x	5000	0.174	0.383	0.141	0.055	0.214	0.214
		10,000	0.172	0.393	0.122	0.054	0.211	0.211
2	R50-FPN 3x	5000	0.23	0.505	0.164	0.058	0.26	0.283
		10,000	0.231	0.524	0.17	0.054	0.272	0.288
3	R101-DC5 3x	5000	0.237	0.59	0.133	0.045	0.27	0.324
		10,000	0.237	0.569	0.155	0.042	0.271	0.33

## Data Availability

The data presented in this study are openly available in GitHub https://github.com/neclipse/Metal_defects_detection/tree/main.

## Supplementary Materials

The following are available online at https://github.com/neclipse/Metal_defects_detection/tree/main/Supplementary%20Materials, Figure S1: The original table of level one CNN model summary for Figure 1, Figure S2: The original total loss curve for Figure 8, Video S1: The original video before testing for Figure 11a, Video S2: The original output video using Detectron2 model for Figure 11b.
